# Real time monitoring of membrane GPCR reconstitution by plasmon waveguide resonance: on the role of lipids

**DOI:** 10.1038/srep36181

**Published:** 2016-11-08

**Authors:** Pierre Calmet, Monica De Maria, Etienne Harté, Daniel Lamb, Maria Serrano-Vega, Ali Jazayeri, Nuska Tschammer, Isabel D. Alves

**Affiliations:** 1 Max Planck Institute for the Science of Light, Erlangen, Germany; 2 Friedrich Alexander University (FAU) Erlangen-Nürnberg, Erlangen, Germany; 3 Chemistry and Biology of Membranes and Nanoobjects, UMR 5248 CNRS, University of Bordeaux, Bat. B14 allée Geoffroy St. Hilaire, 33600 Pessac, France; 4 Department of Developmental Biology, Friedrich Alexander University of Erlangen-Nürnberg, Erlangen, Germany; 5 Heptares Therapeutics Ltd, BioPark, Broadwater Road, Welwyn Garden City, Hertfordshire AL7 3AX, UK; 6 NanoTemper Technologies GmbH, Munich, Germany

## Abstract

G-protein coupled receptors (GPCRs) are important therapeutic targets since more than 40% of the drugs on the market exert their action through these proteins. To decipher the molecular mechanisms of activation and signaling, GPCRs often need to be isolated and reconstituted from a detergent-solubilized state into a well-defined and controllable lipid model system. Several methods exist to reconstitute membrane proteins in lipid systems but usually the reconstitution success is tested at the end of the experiment and often by an additional and indirect method. Irrespective of the method used, the reconstitution process is often an intractable and time-consuming trial-and-error procedure. Herein, we present a method that allows directly monitoring the reconstitution of GPCRs in model planar lipid membranes. Plasmon waveguide resonance (PWR) allows following GPCR lipid reconstitution process without any labeling and with high sensitivity. Additionally, the method is ideal to probe the lipid effect on receptor ligand binding as demonstrated by antagonist binding to the chemokine CCR5 receptor.

G-protein coupled receptors constitute the largest protein family in the human proteome (over 800 members). Their diversity and central role in cellular signaling makes them important drug targets. Indeed, GPCRs are the targets of about 40% of all therapeutic drugs, including 25% of the top-selling ones^1^. Understanding molecular mechanisms of GPCR signaling is of both fundamental scientific impact and high medical potential. Biophysical studies on these receptors are particularly challenging because they are transmembrane proteins that require the appropriate hydrophobic lipid environment for proper function. Following their isolation from cellular membranes using detergents that preserve their function, reconstitution in lipid membranes is necessary to maintain their stability. Their reconstitution from a detergent-stabilized state into a well-defined artificial lipid model system of controlled lipid composition for *in vitro* studies^2^,^3^ can be achieved by dilution or detergent removal using dialysis, gel filtration, hydrophobic absorption using Bio-beads, or complexation by cyclodextrins. Regardless of the method used, the process itself remains most often intractable (with the exception of an indirect Isothermal Titration Calorimetry approach reported by S. Keller and coworkers based on phase transition monitoring^4^). Herein we demonstrate that Plasmon Waveguide Resonance (PWR) spectroscopy can be used to directly follow the reconstitution of a detergent-solubilized GPCR into a planar solid-supported lipid membrane^5^. The method can be extended to any detergent-purified membrane protein, considering that the detergent used does not induce large perturbations or partial destruction of the lipid membrane where the protein is to be reconstituted.

The GPCR under study, the CC chemokine receptor 5 (CCR5), is expressed in the immune system by macrophages, dendritic cells, NK, NKT and T cells, where it triggers T-cell activation, regulates inflammation, and directs chemotaxis. This receptor is best-known as the principal co-receptor for entry of HIV type 1 (HIV-1), used together with CD4 to enter and infect target cells^6^.

To ease the purification and further manipulation of the protein, a thermostable variant of CCR5 (referred to as CCR5 StaR) has been used. This construct has been stabilized in the antagonist conformation as described previously (manuscript in preparation)^7^. Because the protein is stabilized in the antagonist conformation, we focused our analysis on the allosteric inhibitor maraviroc, one of the first discovered anti HIV drugs, as a test ligand for the study^8^,^9^,^10^,^11^.

The spontaneous insertion of the receptor into the lipid membrane through the so-called detergent-dilution reconstitution method can be monitored directly by PWR thanks to its sensitivity to changes in mass density. Additionally, PWR can measure anisotropy changes in oriented system such as lipid bilayers, and therefore confirm the proper orientation in the membrane of GPCRs and other anisotropic structures. Detailed kinetic information along with respective rate constants is obtained, yielding mechanistic information about the reconstitution process. Moreover, PWR allows for an immediate confirmation of the functionality of the inserted protein, by monitoring ligand-induced conformational changes that reflects the activity of the reconstituted receptor. The added value of this technology resides on the fact that both receptor reconstitution and activity (ligand binding or G-protein binding) can be evaluated in a single experiment in real time, directly (no labeling required) and with high sensitivity (femtomole quantities of receptor can be detected). Methods often employed do not allow for both aspects to be investigated. Commonly, reconstitution of membrane proteins in different lipid model systems (lipid vesicles, bicelles, nanodiscs) is confirmed after the reconstitution process is over by SDS-PAGE and Western-blot or by biophysical methods (NMR, infrared and Raman vibrational spectroscopy, electron paramagnetic resonance, circular dichroism, and fluorescence spectroscopy) following separation of reconstituted systems from non-reconstituted ones (by sucrose gradients or other purification methods). Alternatively fluorescence microscopy or other imaging methods are used to analyze reconstituted protein in giant unilamellar vesicles (GUVs), which requires labeled material. Following that, the activity of the protein is measured in yet a different experimental setup. Overall the whole procedure is tedious, long and requires often high quantities of material and the use of labeled proteins. PWR provides a good alternative with the only limitation being that the detergent in which the protein is solubilised must not perturb the membrane integrity. Care must be taken with detergents possessing a low critical micelle concentration (cmc) value, like dodecylmaltoside, often employed for membrane protein solubilisation. In this case there are two alternatives: 1) to use very concentrated protein solutions for the reconstitution process to minimize the amount of detergent added 2); to exchange the detergent before reconstitution with a detergent with a higher cmc value like octylglucoside.

Although the growing number of GPCR X-ray structures, most in ligand-bound state, has been crucial for the understanding of their action mode, the role of the lipid environment both in the activation and first signaling events received less attention. The GPCR that has received the most attention in that matter is rhodopsin where lipids such as phosphatydylethanolamine (PE) were reported to promote the metharhodopsin MI-MII transition (from the basal to the light-activated state of rhodopsin) and even transducin (the rhodopsin G protein) binding^12^,^13^,^14^. Rod outer segment membranes are enriched in PE, and predominantly express rhodopsin which represent its main protein component (over 90%). This unique receptor-lipid relationship has most likely evolved to support the function of rhodopsin. The influence of the lipid environment on the functions of other GPCRs is less established, but a more complicated picture is expected, as the membranes where other GPCRs are expressed are much more diverse in both protein and lipid composition. In particular, this could shed light on the importance of lipid and protein segregation in the membrane.

This aspect in part motivated the study of lipid modulation of CCR5 ligand-binding activity presented here. Although the maraviroc-bound CCR5 high resolution structure was recently determined^15^, the role of lipids in the receptor function remains obscure. Lipid domains rich in cholesterol and sphingomyelin (so-called lipid rafts) have been shown to modulate the activity of several GPCRs^16^,^17^,^18^,^19^. Lipid rafts may work as recruiting platforms to locally increase the concentration of receptor and effector molecules selectively activating specific signaling cascades^20^. In addition, the ordered lipid environment provided by rafts may affect functional properties of the receptors. Chemokines have been shown to preferentially bind to receptors associated with cholesterol and sphingomyelin-rich lipid microdomains^21^. It was shown that CCR5 requires membrane cholesterol to retain ligand binding and signaling functions. Studies have indicated that cholesterol is required for the entry of HIV virions into cells expressing the co-receptors CCR5 and/or CXCR4, and that cholesterol depletion inhibits HIV entry^22^. It has also been revealed that cholesterol is important for chemotaxis of human neutrophils, but has no influence on early signaling by chemokine receptors on those cells^23^. The implication of rafts in the activation of CXCR4 by CXCL12 has also been shown in the context of prostate cancer^24^. Furthermore, cholesterol depletion leads to failure of CCR5 to inhibit cAMP activity^25^; lipid action on the receptor can therefore be present both at the level of the ligand but also at effector interactions, affecting receptor downstream signaling, internalization and desensitization. Although the general consensus that cholesterol influences the chemokine receptor function has been reached, the answer on how and at which level (receptor ligand binding affinity, kinetics, conformational change, interaction with downstream signaling partners) this takes place is still contradictory. The source of this contradictions might lie in the fact that the reagents used for the cholesterol depletion in living cells might *per se* cause artefacts as reported for methly-β-cylodextrin^26^. We therefore propose a bottom-up approach where we strictly control the lipid composition. PWR along with the use of planar lipid bilayers of controlled lipid composition is ideal to follow the role of lipids in membrane protein activity. PWR studies in lipid membranes composed of palmitoylphosphatidylcholine (POPC), POPC and cholesterol (POPC/Chol; 8/2 mol/mol), and a mixture of the previous with sphingomyelin and (POPC/Chol/brain SM; 6/2/2 mol/mol/mol; as mimic of lateral heterogeneous membranes, with rafts) presented here determined the role of lipid domains and that of cholesterol alone in both CCR5 StaR reconstitution kinetics, maraviroc-CCR5 StaR binding affinity and magnitude of ligand-induced conformational changes in the receptor.

## Results

### Reconstitution of CCR5 StaR in a planar lipid membrane

**Figure 1:**
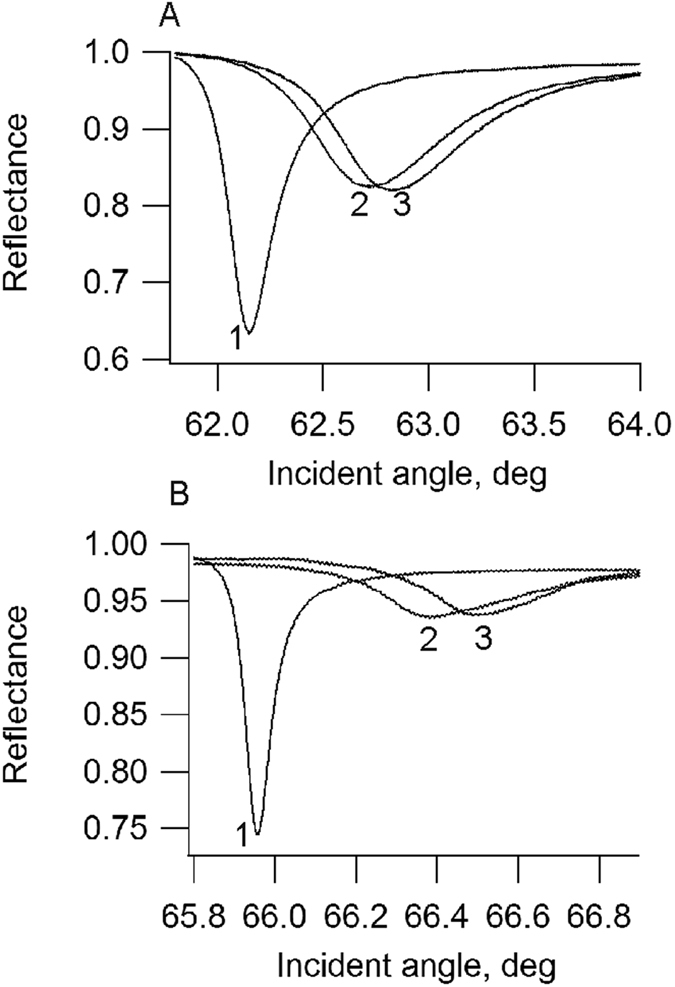
PWR spectra of the buffer (1), a POPC lipid bilayer (2) and after reconstitution of CCR5 StaR (3) in the membrane, obtained with *p*- (**A**) and *s*- (**B**) polarization.

PWR was used to follow CCR5 StaR insertion into planar lipid membranes composed of POPC, POPC/Chol (8/2 mol/mol) and POPC/SM/Chol (6/2/2 mol/mol/mol). The method is sensitive to mass gain in the lipid bilayer that results from protein insertion, to changes in the bilayer thickness and to changes in anisotropy measured by following spectra changes with both *p*- and *s*-polarizations. The addition of the 20 μL octylglucoside-solubilized CCR5 StaR to the lipid bilayer (about 1 μM of protein) resulted immediately in an increase in the resonance minimum position for both polarizations. This arises from a mass gain in the system leading to an increase in the refractive index. The reconstitution process was followed until completion indicated by no further changes in the resonance position which takes about 1–2 hours. Following that, the cell sample compartment is washed to remove superficially-bound receptors that were not properly inserted in the membrane and any protein that remained in the bulk solution. This resulted in small decreases in both resonance angles as well as on the TIR angle that is particularly sensitive to the optical properties of the material that is in the bulk, so farthest away from the sensor surface (see Experimental section for details). The reconstitution process (after washing) produced resonance spectral shifts of about 110 and 80 millidegrees (mdeg) for *p*- and *s*-polarizations, respectively (Fig. 1). The spectral depth also increased upon receptor incorporation, a property that relates to changes (in this case increase) in the thickness of the lipid film. The increase in membrane thickness following receptor incorporation can be explained by the fact that GPCRs have extramembrane parts on both sides of the bilayer and so are thicker than a membrane. An alternative explanation is that the hydrophobic core of the membrane stretches to accommodate the hydrophobic transmembrane domain of the protein leading to an increase in lipid ordering. Additionally, one should mention that the spectral shifts were larger for *p*- than *s*-polarization, this is very important since as GPCRs are anisotropic molecules (described by an ellipsoid that is ~75 Å perpendicular to the membrane, ~48 Å wide in the standard view, and ~35 Å thick for the model GPCR rhodopsin^27^), it indicates that the reconstituted receptor is oriented with the long-axis parallel to the *p*-polarized light (or perpendicular to the membrane) and the short axis parallel to the membrane as it is found in nature. In what concerns the direction of orientation of CCR5 StaR in the membrane, we cannot determine the percentage of receptor facing its extracellular side towards the buffer compartment accessible to ligand binding. We know from the fact that the receptor binds ligand (next section) that some receptor molecules must have the extracellular side facing the buffer compartment. 1D spectral simulation confirmed that the protein is anisotropic. Its long axis has a refractive index of 1.54 and its short axis of 1.45. The long axis is oriented perpendicularly to the membrane plane. The average thickness of the protein layer was calculated from simulation. The average thickness of the protein layer comprises both membrane without protein which we have determined to be about 4.5 nm and membrane with protein inserted that we estimated based on the thickness of rhodopsin to about 7.5 nm. Our calculation indicated the average proteomembrane thickness to be 4.6 nm. This value is smaller than the long axis of the protein (reported to be 7.5 nm). This implies a partial membrane protein coverage. To calculate the mass of protein deposited per unit surface, we calculate L, the number of moles per volume with the following equation:


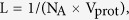


where N_A_ is the Avogrado number and Vprot is the volume of the protein (approximated to a cylinder of 4.8 nm diameter and 7.5 of length). The mass density of the protein can be calculated using the equation:


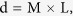


where M is the molecular weight of the protein. Knowing the thickness of the protein layer, the incorporated mass of the protein is evaluated at 22 μg/cm^2^ that corresponds to a P/L ratio of 1/110 (about 50% of membrane coverage). This corresponds to about 1/6th of the added protein to the PWR cell sample, being reconstituted in the membrane. The addition of the same concentration of protein-free detergent to a similar membrane system had a very slight effect on resonance positions (+2 mdeg for the resonance observed with *p*-polarization, +1 mdeg for the resonance observed with *s*-polarization, +2 mdeg for TIR angle, data not shown), which faded upon washing with buffer (+1 mdeg for the resonance observed with *p*-polarization). Therefore, the low concentration of detergent negligibly affects the properties of the membrane, cannot account for the large shifts observed. This ensures that the kinetics observed by PWR after protein addition cannot be attributed to detergent incorporation, but to protein reconstitution.

The reconstitution of the receptor in a solid-supported lipid membrane was also observed by polarized ATR-FTIR that thanks to the specific IR absorption of lipids and proteins allows their discrimination. Moreover by measuring the spectra with both p- and s-polarized light the orientation of the lipid bilayer and that of the protein can be determined^28^. Additionally since the amide I band position depends on the type of secondary structure adopted by the protein, an estimation of the secondary structure of the protein is possible. The addition of the octylglucoside-solubilised receptor to the solid supported membrane formed in the germanium crystal leads to the appearance of an amide I signal indicating that the protein is inserted in the membrane. The maximum of the amide I absorption peak spans from around 1640 to 1660 meaning that there is both helical and random coil structures in the protein. The dichroic ratio (R_ATR_) obtained for the amide I region was about 2, meaning that the helices are oriented with their long axis perpendicular to the membrane (Fig. S1 in SI). This agrees with the results observed by PWR.

**Figure 2:**
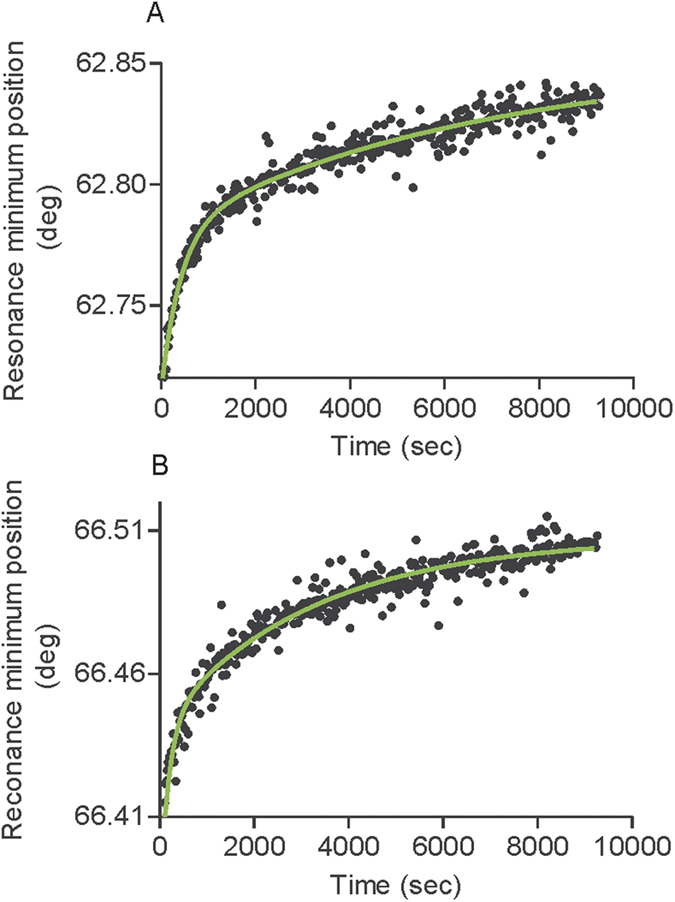
Kinetic data for CCR5 StaR reconstitution in a POPC lipid membrane obtained by PWR with *p*- (**A**) and *s*- (**B**) polarization. The data was analyzed with Graph Pad Prism using a two phase exponential association equation (The fit is shown in green; more details can be found in Materials and Methods). Rate constants obtained for this and other lipid systems are presented in Fig. 3 and in Table 1.

PWR spectra were recorded over time in order to investigate the kinetics of reconstitution. The best fit was obtained using a two phase exponential model which postulates that receptor reconstitution is the result of the sum of two exponential decay (details can be found in Experimental section) with a fast and a slow rate constant (Fig. 2, Table 1). It is commonly used to describe a reaction whose rate is proportional to the amount of one reactant. It is expected to be the case if we consider the limiting step of the reconstitution to be the binding.

**Figure 3:**
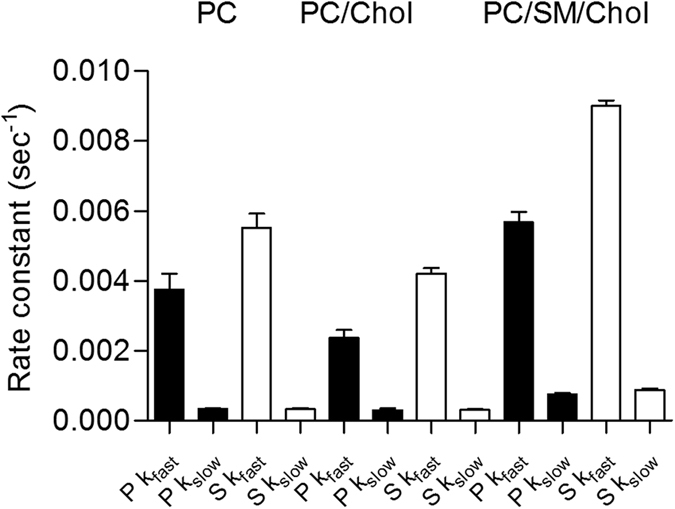
Rate constants obtained with *p*- and *s*-polarized light for the reconstitution of CCR5 StaR into a lipid membrane composed of POPC, POPC/chol (8/2 mol/mol) and POPC/SM/Chol (6/2/2 mol/mol/mol). The reconstitution process follows a two-stage exponential process with two rate constants associated (fast and slow) (see Materials and Methods for details). Black and white bars correspond to the data obtained with *p*- and *s*-polarized data, respectively. Data in bars correspond to the mean value and associated standard deviation obtained from at least 4 independent experiments. Significativity of the difference between the values is presented in Table SII.

One can propose that the two phases correspond to receptor insertion followed by changes in orientation. Analysis of the data provided fast (k_fast_) and slow (k_slow_) rate constants for both the *p*-pol and *s*-pol data. Overall rate constants for the fast process were higher for *s*- than *p*-polarization, which may reflect fluctuations in the protein tilting and anisotropy (Fig. 2, Table 1). The kinetics of the reconstitution process was significantly affected by the membrane lipid composition. Reconstitution was significantly slower in membranes containing cholesterol (POPC/Chol) than in pure POPC membranes (see Fig. 3, Table 1 and SI for statistical analysis of significance). On the contrary, the reconstitution was faster in laterally heterogeneous membranes (composed of PC/SM/Chol).

### Response of CCR5 StaR to maraviroc

**Figure 4:**
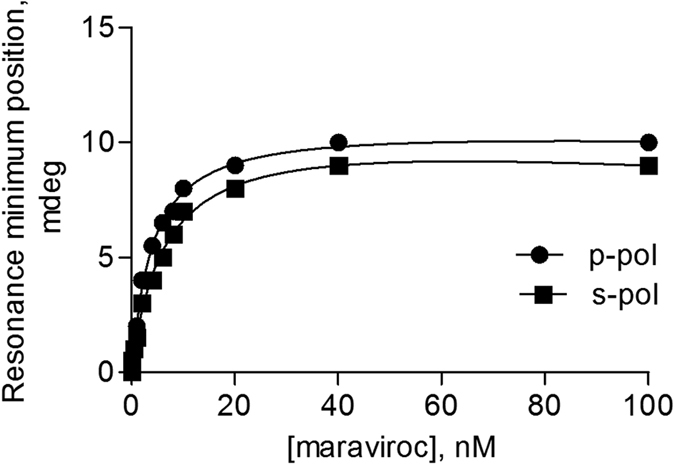
Ligand interaction with CCR5 StaR reconstituted in a POPC membrane monitored by PWR. The ligand maraviroc was incrementally added to the proteolipid membrane and the shifts in the resonance minimum position followed. The data was fitted with a hyperbolic binding equation that describes total binding to a single site in the receptor (more details in Materials and Methods). Dissociation constants obtained from the data are presented in Table 2.

To test CCR5 StaR functionality after lipid reconstitution, the capacity of the receptor to bind ligand was tested. The addition of maraviroc increased the resonance position for both polarizations (Fig. 4). It should be noted that maraviroc addition to a lipid bilayer with no receptor incorporated, resulted in no spectral changes, thus indicating that the response observed is related to the receptor (data not shown). Moreover, as maraviroc is a small molecule, the binding alone of maraviroc to the receptor could not explain the spectral changes observed as the mass of the ligand itself is too small to account for the observed spectral changes. Thus these spectral changes cannot arise from the binding of maraviroc itself to the proteolipid system but from the receptor conformational changes induced by ligand binding. Additionally the binding results in a hyperbolic saturable response that is typical of ligand binding to a receptor. Calculated dissociation constants (K_D_) from the data provide values of about 4 nM for the interaction of maraviroc with CCR5 StaR reconstituted in a POPC membrane (See Fig. 4 and Table 2). This value is in good correlation to what has been reported in the literature obtained in cellular systems, for the binding of maraviroc to CCR5^29^. As elucidated from the high resolution X-ray structure, maraviroc binds in the bottom of a pocket defined by residues from helices I, II, III, V, VI and VII^15^. Thus it implies that every transmembrane helix, except helix IV are implicated in binding. The recognition of maraviroc by the receptor implies that the receptor is properly folded and biologically active after reconstitution. Moreover, the binding affinity of maraviroc to the detergent solubilised receptor determined by competition radiolabeling experiments was found to lie between 3 and 100 nM (Fig. S2 in SI), again an affinity that correlates to that obtained after the reconstitution process. The affinity is increased by a factor of 4 when the membranes include cholesterol (either with or without sphingomyelin).

## Discussion

### PWR as a method to follow GPCR reconstitution and receptor functionality

There are different methods for the reconstitution of detergent-solubilized membrane proteins, most common are detergent-dilution, dialysis methods or hydrophobic absorption (by the use of Bio-beads) (see ref. 30 for a review). A more recent method based on charge-interaction directed reconstitution mechanism has been reported for the spontaneous insertion of bovine rhodopsin in both lipid- and polymer-based artificial membranes^31^. A preferential contact between a membrane protein hydrophilic domain and the membrane by charge attraction triggers membrane protein insertion and decortications of detergent micelles associated with the membrane protein hydrophobic domain.

While protein reconstitution processes can be checked at different stages by the use of different spectroscopic techniques (often light scattering and turbidimetry) to ensure that the reconstitution is proceeding correctly or by the end by SDS-PAGE or Western Blot analysis, these measurements are quite indirect and cannot ascertain proper protein insertion nor protein activity. Indeed, the reconstitution process success and protein activity after reconstitution is most often checked afterwards, by measuring the functionality of the reconstituted protein by *in vitro* biophysical approaches such as NMR^32^,^33^,^34^,^35^, infrared^36^ and Raman vibrational spectroscopy^37^, electron paramagnetic resonance (EPR)^38^, circular dichroism^15^,^33^,^34^,^35^, and fluorescence spectroscopy^39^,^40^.

Very few methods exist to follow the reconstitution of GPCRs in lipid model systems in a direct manner (no labeling required) and as far as we know no method allows one to obtain rate constants for this process. Dual-color fluorescence cross-correlation spectroscopy (FCCS) has been reported of great value for optimizing membrane protein reconstitution processes thanks to its ability to distinguish micelles, liposomes and aggregates in heterogeneous mixtures and by the fact that it allows co-localization of proteins and lipids in the diffusing assemblies^41^. The disadvantage of the method is its reliance on fluorescence, which requires fluorescent labelling of the protein and the lipids, and limits the time resolution to avoid bleaching. Yet, another approach reported by Keller and collaborators allows monitoring the process the phase changes concurrent with the reconstitution^4^. While the method is ideal to optimize the conditions of detergent-mediated reconstitution, it still relies on the use of additional techniques to confirm proper protein reconstitution and functionality.

PWR offers the advantage of directly following the insertion and orientation of detergent-solubilized membrane proteins in planar lipid membranes. Detergent dilution below the detergent cmc allows spontaneous insertion of the protein in the membrane and washing the cell sample compartment allows excess of detergent and protein to be removed. Additionally it provides rate constants for the process and the possibility of testing receptor functionality within the same experimental setup, with the same sample. Moreover due to the sensitivity of the method, it does not require high amounts of protein which is an important aspect to consider when working with GPCRs. Certainly the method suffers from some disadvantages: (1) it is not compatible with the use detergents that tend to destroy lipid membranes at low concentrations such as those possessing a low cmc (like DM and DDM). Unfortunately those are the most commonly used detergents for receptor solubilisation. To deal with this problem, the detergent can be diluted in a membrane-friendly detergent such as octylglucoside (this is the approach used here). Alternatively, the detergent can be exchanged right before reconstitution; (2) It does not ascertain that all detergent molecules are removed from the proteolipid system by washing. Although, so far if detergent molecules were present the binding activity studied by PWR of several GPCRs reconstituted in lipid membranes was not affected^42^,^43^,^44^; (3) Receptor insertion direction is not controlled and cannot be directly determined. This can be a disadvantage or an advantage. Disadvantageous if every protein is inserted in the “wrong” orientation, for a GPCR that would be with the intracellular side facing the buffer solution and so unable to recognize ligand. It can be advantageous if the protein is inserted in two different populations, intracellular side or extracellular side facing the buffer solution, and if one is testing both ligand and G-protein binding. In that case the receptor can be pre-incubated with ligand before reconstitution and then the effect of ligand tested on the receptor ability to bind G-proteins^45^.

PWR can also be used to investigate the activity of membrane proteins reconstituted in nanodiscs (unpublished data) and of membrane proteins embedded in supported lipid bilayers formed by the fusion of small unilamellar vesicles (SUVs) already with the reconstituted protein. These two options are attractive when proteins are extracted from cells along with their native lipid environment, or when the membrane protein reconstitution procedure calls for controlled detergent removal using Bio-beads. In those cases the reconstitution of the receptor cannot be followed by PWR because the reconstitution process is performed a priori but the protein activity can be followed in the same manner as what has been described in this work. Since the orientation of the protein is also kept in those model systems, information about the anisotropy of the system is also obtained.

Herein we have shown to be able to follow octylglucoside-solubilized CCR5 StaR reconstitution in planar lipid membranes. Kinetic data indicates that it follows a two-state process with a fast phase occurring within around 5 minutes followed by a slow phase occurring in over 30 minutes. From the kinetics and spectral changes occurring in both polarizations and the TIR, we can attribute the first stage to receptor insertion in the lipid membrane and the second stage to the adoption of a proper orientation (with the long axis perpendicular to the membrane). The functionality of the reconstituted protein was confirmed by its capacity to bind to the antagonist maraviroc. The measured affinity of maraviroc for CCR5 StaR resembles that reported by cellular studies on binding to wild type CCR5^29^. This demonstrates that the reconstituted receptor is properly folded and functional. Moreover, it indicates that mutations introduced into CCR5 to render the receptor thermostable do not affect maraviroc binding affinity.

### Role of cholesterol in CCR5 StaR reconstitution and ligand binding activity

Due to the reported possible role of cholesterol and that of cholesterol and sphingomyelin-rich lipid domains (rafts) in CCR5 signaling, as presented in introduction section, we have followed both CCR5 StaR reconstitution and ligand response in membranes in absence and presence of cholesterol and in presence of cholesterol and sphingomyelin. We were surprised to find that the presence of cholesterol in POPC membranes (POPC/Chol 8/2 mol/mol) affected the reconstitution process by slowing it down to almost half speed. Reconstitution in POPC/SM/Chol (6/2/2) membranes was about two times faster than that observed in POPC. For POPC/chol membrane this could be related with the fact that cholesterol increases the order of the POPC fluid membrane rendering it less prone to receptor insertion. The increase in the reconstitution rate taking place in POPC/SM/Chol membrane might be due to the fact that membranes with lateral heterogeneity present places of low surface tension or defects (in the contacts between the different domains) that can be opportunistically used by the receptor to insert in a facilitated manner^46^. Alternatively it is also possible that the receptors inserts faster in brain SM rich domains that have increased thickness compared to disordered domains that might better match the receptor hydrophobic portion. Different domain partition of GPCRs has been reported^16^,^20^,^47^,^48^. The sole reported study on the role of cholesterol in membrane protein reconstitution demonstrated that cholesterol catalyzes insertion of bacteriorhodopsin, UDPglucuronosyltransferase and cytochrome oxidase^49^ in liposomes. What differs in this study compared to the present work is that the proteins were reconstituted in lipids that were in the gel phase (DMPC) at the temperature at which experiments were performed, so cholesterol lowered the energy barrier for protein reconstitution by fluidizing the membrane while our studies where performed with membranes in the fluid phase (POPC) and so cholesterol had an ordering effect.

The binding activity of CCR5 StaR was also affected by the presence of cholesterol. Indeed the presence of cholesterol led to about 4 fold increase in the affinity of maraviroc to the receptor. This could be due to the different physico-chemical properties of the membrane in presence of cholesterol. The magnitude of the spectral changes (shifts in the resonance minimum position in mdeg) is a measure of the magnitude of the mass and structural changes occurring in the proteolipid system. The magnitude of the conformational change is also directly related to the amount of receptor incorporated in the membrane. Here we have taken that into account and to correct for that, the data was normalized to the amount of reconstituted receptor (directly related to the magnitude of the spectral shifts observed after protein reconstitution relative to the membrane alone). Since the magnitudes of the ligand-induced PWR spectral changes are very much influenced by the presence of cholesterol in lipid membranes (less than half the shift for *p*-pol and 6–9 times smaller for *s*-pol when cholesterol is present) this can result from a different receptor conformational change induced by the ligand when the protein is reconstituted in different lipid systems. Since in the studies both laterally homogeneous lipid membranes and membranes composed of ordered and disordered lipid domains were employed, and the effect was similar in both cases, it means that the response comes from cholesterol itself independently of its location in the membrane. As CCR5 is known to localize in cholesterol enriched regions in cells, segregation of CCR5 in cholesterol-rich domains could therefore gate this regulation by cholesterol. This is of particular significance in a cellular context, where the localization of receptors can have a functional role in signalization, for example in the case of a polarized cell.

The presence of cholesterol has been shown essential for the interaction between CCR5 and HIV gp120 occurring during viral infection, as their depletion from cells inhibits viral infection and reloading cholesterol restores the process^50^. Cholesterol depletion in cells by β-methyl cyclodextrin has been reported by Nguyen and Taub^51^ to inhibit CCR5 receptor activation by CCL4, the endogenous agonist for this ligand. Similar results have been obtained by Marsh and co-workers, where cholesterol sequestration by filipin was shown to interfere with agonist binding to CCR5^52^. While strong evidence exists for cholesterol effect on agonist-mediated activation of CCR5, the role of cholesterol in antagonist binding has not been investigated. Moreover, because of the type of approaches used in those studies, it is not possible to determine if the effect results from the cholesterol molecule itself or from the presence of lipid domains rich in cholesterol and other lipids as sphingomyelin (called lipid rafts). Our studies shed light into this unexplored aspect and demonstrate that cholesterol effect comes from the cholesterol molecule itself that induced a different antagonist-induced conformational change in CCR5. Yet, an aspect that remains unanswered is whether cholesterol effect in the antagonist-induced receptor conformational change is a pure result of the alterations in the physical properties of the membrane (e.g., fluidity) or by a direct binding to the CCR5 receptor or a combination of both. Indeed, a putative-cholesterol binding site has been identified in human CXCR4 by docking analysis and in CCR5 by sequence alignment^53^ and cholesterol was even used in CXCR4 crystallization^54^.

## Methods

### Chemicals

Lipids were purchased from Avanti Lipids. Salts, buffers and solvents were purchased from Sigma-Aldrich. The ligand maraviroc was from Tocris and ^3^H-Cpd1 and Cpd1 were from Key Organics. The detergent-solubilised receptor CCR5 StaR was provided by Heptares.

### Plasmon waveguide resonance (PWR)

PWR spectra were obtained with a home-made instrument equipped with a He-Ne laser at 632 nm whose light is linearly polarized at 45°, allowing acquisition of both *p*- (TM component of light; light that is parallel to the incident light) and *s*-polarization (TE component of light; light that is perpendicular to the incident light) data within a single angular scan^5^. Experiments are performed in a controlled temperature environment of 23 °C. The sensor consists in a 90° angle prism whose hypotenuse is coated with a silver layer (50 nm) and overcoated with silica (460 nm). The prism is in contact with the cell sample Teflon block, with an aperture of approximately 3 mm through which the lipid bilayer is formed. This is placed on a rotating table mounted on a corresponding motion controller (Newport, Motion controller XPS; ≤1 mdeg resolution) so that the incident light angle is changed by steps of 1 mdeg to search for the resonance angle. The reflected light as a function of the incident light is detected by a photodiode (Hamamatsu) and registered on a photodiode amplifier (Thorlabs).

Within a scan of less than 10°, both *p*- and *s*-polarization resonances are obtained with features that reflect the properties of the immobilized interacting molecules, namely, resonance angle minimum position, spectral depth and width. Once the resonances have been located, kinetics in the millisecond scale can be acquired with this instrument, by focusing on the angular region of the resonances rather than acquiring the full spectra. Such resonance properties are sensitive to the optical properties of the materials (refractive index *n* and extinction coefficient *k* for both *p*- and *s*-pol; the thickness *t* the deposited material) that are deposited in close proximity to the sensor surface as the evanescent wave produced under resonance conditions decreases exponentially with the distance from the surface. It should be pointed out that the *p*-pol and *s*-pol resonances and total internal refection angle (TIR) respond differently as a function of the distance from the sensor surface (see Fig. S3 in Supplementary Information). Indeed, theoretical simulation studies by our laboratory have demonstrated that sensitivity to the thin and bulk sample varies between *s*-pol, *p*-pol and TIR. Consequently, an isotropic refractive index variation leads to different ratios of angular shifts for the TIR, *s*-pol and *p*-pol for a thin or a bulk sample as described in Table 1 (Supplementary information). Thus, it is possible to determine from this ratio if the change is localized to the surface or if it’s affecting the whole volume of the sample.

For example, when adding a molecular partner to the PWR cell with a considerably different refractive index from that of the system, there is automatically a change in the resonance position of both *p*- and *s*-polarizations and TIR. Then if all molecules added bind to the surface of the prism, the TIR will return to its initial position while *p*- and *s*-polarizations resonances will evolve according to the change of mass density and anisotropy induced by the binding. Upon saturation of the system with the molecular partner, the TIR starts to increase again, reflecting the excess of material in the bulk phase.

The instrument is controlled via a program written with Labview (National Instruments) that is also used to streamline data analysis. Thus, aside from the spectra itself, properties that characterize a spectra as the resonance minimum position for *p*- and *s*-pol, the TIR angle, the spectral depth for both polarizations were recorded and monitored directly during the experiment. Additionally, the diagrams correlating the different parameters can also be obtained, which can aid in a first level data interpretation, for example a correlation of the resonance shifts obtained for *p*- and *s*-polarized light can directly provide information on the anisotropy of the response.

In terms of data analysis, quantitative information on the affinity (anywhere from pM to mM) or kinetics (msec) can be obtained (this is further described in sections 2.4 and 2.5). Another type of analysis is possible that consists in spectral simulation to obtain all optical parameters that characterize a PWR spectrum: refractive index and extinction coefficient for *p*- (*n*_*p*_ and *k*_*p*_, respectively) and *s*-pol (*n*_*s*_ and *k*_*s*_, respectively), thickness (*t*) of the deposited thin film. Fits of experimental spectra were performed with Winspall software which is based on the Fresnel equations and the matrix formalism (Worm J., WINSPALL, version 3.02; 2009; http:/www2.mpip-mainz.mpg.de/knoll/soft).

### Formation of a planar lipid membrane

The planar lipid bilayer is formed across a small hole (~3 mm diameter) in the Teflon PWR cell sample (total volume of the cell sample compartment is 280 μL). The method employed to form the bilayer is based on the method used by Mueller and Rudin in the 70’s where a lipid bilayer is painted across a small hole in a Teflon block^55^. To do so, about 2.5 μL of a 10 mg/mL solution of lipid (palmitoyloleoilphosphatidylcholine, POPC; POPC/brain sphingomyelin/cholesterol 6/2/2 mol/mol/mol and POPC/cholesterol 8/2 mol/mol) in BuOH/MeOH/squalene (9.45/0.5/0.05 v/v/v) is used. After painting the prism surface with this solution where the lipid headgroups orient towards the hydrophilic silica surface (formation of the first monolayer), the sample is filled with buffer (10 mM Tris-HCl, 100 mM NaCl, 2 mM EDTA, pH 7.4) allowing the formation of the second monolayer. Excess of lipid solution is then removed by washing the bilayer with buffer. To ensure that a proper solid-supported lipid bilayer is formed, the changes in the resonance minimum position (resulting from changes in mass density, anisotropy and thickness following film deposition) for both polarizations are measured and compared to values previously established to correspond to a lipid bilayer^5^.

For polarized ATR-FTIR measurements, the lipid bilayer was formed by spontaneous fusion of small unilamellar vesicles (SUV) of lipid (POPC; POPC/brain SM/chol 6/2/2 mol/mol/mol and POPC/chol 8/2 mol/mol). The total lipid concentration used was 1.5 mg/mL (ca. 1.96 mM). SUV were prepared by tip sonication for 30 min after direct hydration of lipid films with buffer (10 mM Tris-HCl, 100 mM NaCl, 2 mM EDTA, pH 7.4). After bilayer formation at the crystal surface, the excess of SUV was removed by washing with buffer. Since ATR spectroscopy is sensitive to the orientation of the lipid and peptide^56^, spectra were recorded with a parallel (p) and perpendicular (s) polarization of the incident light. All the orientation information is then contained in the dichroic ratio R_ATR_ = Ap/As, where Ap and As represent the absorbance of the considered band for the p or s polarization of the incident light, respectively (for more details see ref. 28).

Polarized ATR-FTIR spectra were recorded on a Nicolet 6700 FT-IR spectrometer (Nicolet Instrument, Madison,WI) equipped with a liquid nitrogen cooled mercury–cadmium–telluride detector (ThermoFisher Scientific, San Jose, CA, USA), with a spectral resolution of 4 cm^−1^ and a one-level zero filling.

### Radioligand Binding activity of the detergent-solubilised CCR5 StaR

The receptor was provided by Heptares that solubilized the receptor in decylmaltoside (DM) and CHS followed by its purification and characterization. Binding activity of the dodecylmaltoside (DDM) solubilised receptor is provided in Supplementary Information (Fig. S2). HEK293T cells transiently expressing CCR5 StaR were solubilised using 1% DDM in 50 mM Hepes pH7.5, 100 mM NaCl. Insoluble material was removed by centrifugation. Cleared lysate samples were incubated at 4 °C for 3 hours with 300 nM ^3^H-Cpd1 (an antagonist for the CCR5 receptor^57^) in the presence of unlabelled competitors indicated (maraviroc and Cpd1) or a DMSO vehicle control. Unbound radioligand was then separated by size exclusion chromatography prior to reading bound radioactivity using a liquid scintillation and a Microbeta counter. Non-specific binding was determined using lysate from untransfected HEK293T cells. In the absence of competing ligand, ^3^H-Cpd1 bound specifically to the CCR5 StaR, but this binding was ablated when either unlabelled Cpd1 or unlabelled Maraviroc were present. These data show that the CCR5 StaR retains the ability to bind structurally diverse small molecule antagonist ligands following detergent solubilisation (Fig. S2 in SI).

### Reconstitution of CCR5 StaR into a lipid membrane

Following lipid bilayer formation and its stabilization, detergent-solubilized CCR5 StaR is reconstituted in the lipid bilayer by the detergent-dilution method. Briefly, 1 μL of the decylmaltoside and CHS-solubilized and purified CCR5 StaR receptor is further diluted in 19 μL of 30 mM octylglucoside (in 10 mM Tris-HCl, 100 mM NaCl, 2 mM EDTA, pH 7.4, same buffer used for all PWR measurements). The addition of considerable amounts of DM to a solid-supported lipid membrane destroys the membrane due to the low cmc of this detergent. Since the protein was purified and stored in DM and CHS and the protein concentration was extremely high we decided to dilute it out in octylglucoside at a concentration just above the cmc. Octylglucoside was used as it a detergent that does not perturb lipid membrane integrity due to is relatively high cmc. That way instead of adding a 1 μL of DM and CHS solubilized CCR5 StaR in a 280 μL cell sample containing the lipid membrane (which is a very small volume that will take more time to diffuse to equilibrium) we decide to dilute out the 1 μL of DM and CHS solubilized CCR5 StaR in 20 μL (final volume) of 30 mM octylglucoside. The addition of 20 μL detergent solubilised receptor into the 280 μL cell sample containing the membrane allows detergent dilution below the cmc and spontaneous insertion of the receptor into the membrane. This procedure is performed right before membrane reconstitution in a way to avoid any effects on protein activity. Following completion of the insertion process (no more changes in the resonance spectra), the excess of CCR5 StaR and detergent from the sample along with detergent is removed by washing the cell sample with at least 3 cell volumes of Tris buffer (3 × 280 μL). Control experiments where protein-free detergent was added to a membrane were performed.

Since the activity of the detergent-solubilised protein was tested in DM and CHS and not in octylglucoside, and to make sure that the short incubation time in octylglucoside prior to reconstitution did not change the protein activity, the reconstitution of of DM and CHS solubilized CCR5 StaR in a POPC bilayer was followed by PWR. Although less amount of protein was incorporated in the membrane (about half relative to the ocylglucoside solubilized protein), probably related to the fact that part of the 1 μL protein volume will denature before reaching the membrane, the protein responded to maraviroc with a similar affinity (Fig. S4). The smaller amplitude of the spectral shifts observed after maraviroc addition are related to the fact that less protein is in the membrane.

For polarized ATR-FTIR experiments, the reconstitution process was performed in the same manner, the only difference being that the chamber is of about 200 μL rather than 280 μL. After incubating the protein with the lipid membrane during 1 h, the excess of detergent and non-inserted receptor is washed out with buffer.

The kinetics data obtained by PWR was fitted by a two phase exponential association process using Graph Pad Prism software (version 5). An exponential equation models many chemical and biological processes. It is used whenever the rate of a reaction is proportional to the amount of one reactant. The two-phase model used postulates that receptor reconstitution is the result of the sum of a fast and slow exponential decay.

### Study of ligand-induced response

Independent CCR5 StaR membrane reconstitution experiments resulted in slightly different shifts in *p*- and *s*-polarized resonances, even when exactly the same amount of protein was added. This then influences the magnitude of the response observed upon ligand addition. In order to be able to compare the data from different experiments, where slightly different amounts of receptor were incorporated into the membrane, the data is normalized.

To study the response of the lipid reconstituted CCR5 StaR to a ligand, maraviroc was added to the proteolipid system in an incremental fashion. Volumes of about 20 μL of the ligand dissolved in the above-mentioned buffer were added to the cell sample, so that after dilution in the cell chamber, the target concentration was achieved. The first concentration point was chosen to be approximately 1 order of magnitude lower than the published dissociation constant (K_D_) value for that ligand. Incremental amounts of ligand were then added in a cumulative fashion, and the PWR spectra were acquired for each point when equilibrium was reached (i.e., when no further changes in the PWR spectra occurred). K_D_ values were obtained from plotting the resonance minimum position for the PWR spectra (this reflects the R-L complex) as a function of total ligand concentration and fitting to the hyperbolic function that describes the 1:1 binding of a ligand to a receptor using GraphPad Prism (GraphPad Software).

A control experiment is performed that consists in adding the same ligand concentrations to a lipid bilayer with no receptor reconstituted. This measures non-specific binding of ligand to lipids alone.

## Additional Information

**How to cite this article**: Calmet, P. *et al*. Real time monitoring of membrane GPCR reconstitution by plasmon waveguide resonance: on the role of lipids. *Sci. Rep.* **6**, 36181; doi: 10.1038/srep36181 (2016).

**Publisher’s note:** Springer Nature remains neutral with regard to jurisdictional claims in published maps and institutional affiliations.

## Supplementary Material

Supplementary Information

## Figures and Tables

**Table 1:** Rate constants obtained with *p*- and *s*-polarized light for the reconstitution of CCR5 StaR into a lipid membrane composed of PC, PC/chol (8/2 mol/mol) and PC/SM/Chol (6/2/2 mol/mol/mol).

Polarization	*p*-pol		*s*-pol	
Phase	Fast (sec^−1^)	Slow (sec^−1^)	Fast (sec^−1^)	Slow (sec^−1^)
POPC	3.8e^−3^ ± 8.8e^−4^	3.3e^−4^ ± 5.9e^−5^	5.5e^−3^ ± 9.1e^−4^	3.3e^−4^ ± 7.5e^−5^
POPC/Chol (8/2)	2.4e^−3^ ± 4.3e^−4^	3.0e^−4^ ± 1.1e^−4^	4.2e^−3^ ± 3.2e^−4^	3.1e^−4^ ± 5.8e^−5^
POPC/SM/Chol (6/2/2)	5.7e^−3^ ± 5.7e^−4^	7.6e^−4^ ± 6.5e^−5^	9.0e^−3^ ± 2.9e^−4^	8.8e^−4^ ± 9.3e^−5^

The reconstitution process follows a two-stage exponential process with two rate constants associated (fast and slow; see Materials and Methods for details). The mean value obtained from at least 4 different experiments and associated standard deviation is provided (95% confidential intervals). Significativity between the different values is provided in SI. Data is presented in the form of bars in Fig. 3.

**Table 2:** Changes induced in the PWR resonance position upon ligand addition to the CCR5 StaR reconstituted in a POPC, POPC/Chol (8/2 mol/mol) and POPC/SM/Chol (6/2/2 mol/mol/mol) and dissociation constants for the receptor/ligand interaction.

	*p*-pol shifts (mdeg)	*s*-pol shifts (mdeg)	K_D_ (nm)
POPC	10 ± 2	9 ± 2	5.3 ± 0.5
POPC/Chol	4 ± 1	1.5 ± 0.5	1.2 ± 0.3
POPC/SM/Chol	3 ± 1	1 ± 0.5	1.4 ± 0.4

Note: The average values and the SD from the mean were obtained from a total of 3 independent experiments.
