# Supplementary material for: Real time monitoring of membrane GPCR reconstitution by plasmon waveguide resonance: on the role of lipids
Source: Sci Rep. 2016 Nov 8;6:36181. doi: 10.1038/srep36181 (PMC5099921; doi:10.1038/srep36181)

Supplementary Material

Real time monitoring of membrane GPCR reconstitution by plasmon waveguide resonance: on the role of lipids

Pierre Calmet[1,2,3], Monica De Maria[4], Etienne Harté[3], Daniel Lamb[5], Maria Serrano-Vega[5], Ali Jazayeri[5], Nuska Tschammer[6], Isabel D. Alves[3*]

[1] Max Planck Institute for the Science of Light, Erlangen, Germany

[2] Friedrich Alexander University (FAU) Erlangen-Nürnberg, Erlangen, Germany

[3] Chemistry and Biology of Membranes and Nanoobjects, UMR 5248 CNRS, University of Bordeaux, Bat. B14 allée Geoffroy St. Hilaire, 33600 Pessac, France

[4] Department of Developmental Biology, Friedrich Alexander University of Erlangen-Nürnberg, Erlangen. Germany

[5] Heptares Therapeutics Ltd, BioPark, Broadwater Road, Welwyn Garden City, Hertfordshire AL7 3AX, UK

[6] NanoTemper Technologies GmbH, Munich, Germany

[*]Corresponding author: email: i.alves@cbmn.u-bordeaux.fr, phone: 0033-5-40006849, fax: 0033-5-40002200

Table SI. Calculated theoretical sensitivity of three sensor modes (TIR, *p*-pol, *s*-pol) for a thin isotropic sample of 5 nm and a continuous isotropic bulk sample.

	Sensitivity (deg/RIU)		
	TIR	*p*-pol	*s*-pol
Thin sample (5 nm isotropic layer)	0	0.58	0.78
Bulk sample	81	59	21

Note: the refractive index of the medium was considered to be 1.33.

Table SII. Significativity between the rate constants obtained for the reconstitution of CCR5 StaR in a POPC, POPC/Chol (1/1 mol/mol) and POPC/SM/Chol (1/1/1 mol/mol/mol). Rate constant data is provided in the manuscript Fig. 3.

Kinetics	Lipids	Polarization		
		p-pol	*s*-pol	*p*-pol *vs* *s*-pol
**Fast**	**PC *vs* PC/Chol**	* P = 0.0308	* P = 0.0286	* P = 0.0216
	**PC *vs* PC/SM/Chol**	* P = 0.0106	*** P = 0.0002	*** P < 0.0001
	**PC/Chol *vs* PC/SM/Chol**	*** P < 0.0001	*** P < 0.0001	*** P < 0.0001
**Slow**	**PC *vs* PC/Chol**	ns	ns	ns
	**PC *vs* PC/SM/Chol**	*** P < 0.0001	*** P < 0.0001	*** P < 0.0001
	**PC/Chol *vs* PC/SM/Chol**	*** P = 0.0003	*** P < 0.0001	ns

Note: Significativity provided by the P values obtained from t-test statistical analysis is calculated from to the data provided in Table I of the manuscript. P values are considered not-significant (ns) when P < 0.05; and from less to more significant with increasing number of *: * for P ≤ 0.05; ** for P ≤ 0.01; *** for P ≤ 0.001.

Figure S1. Polarized ATR-FTIR spectra obtained with p- (blue) and s- (red) polarized light after reconstitution of CCR5 STAR in a POPC lipid bilayer. The amide I and II region of the spectra are shown.

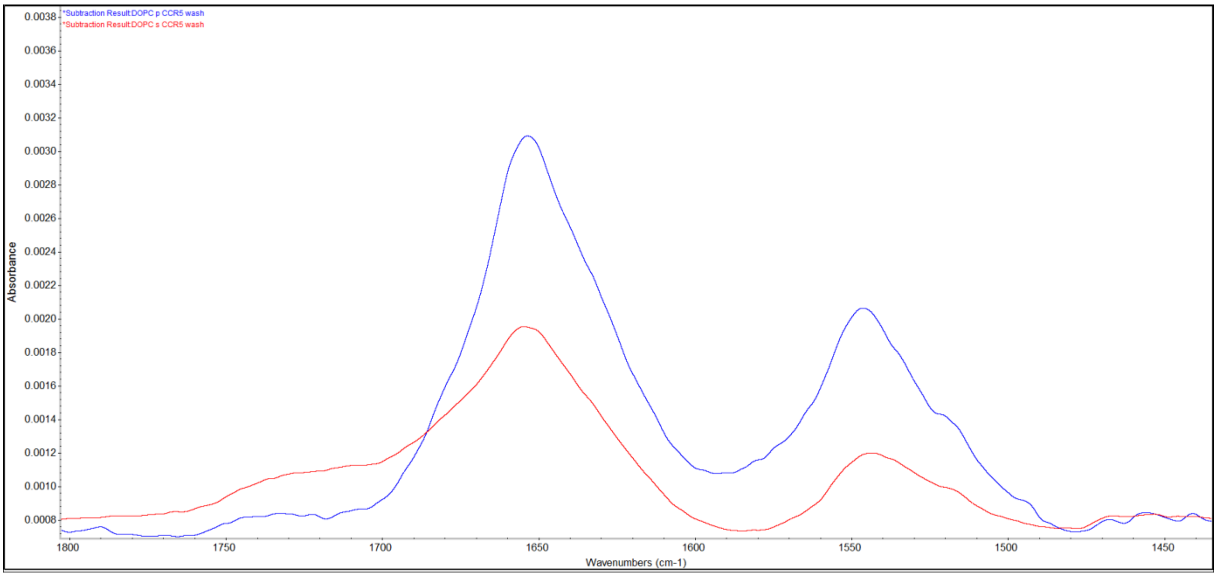

Figure S2. Radioligand binding activity of detergent solubilised CCR5 StaR. DDM solubilised receptor was incubated at 4 ˚C for 3 hours with 300 nM ^{3}H-Cpd1 in the presence of unlabelled competitors indicated or a DMSO vehicle control. Non-specific binding was determined using lysate from untransfected HEK293T cells.

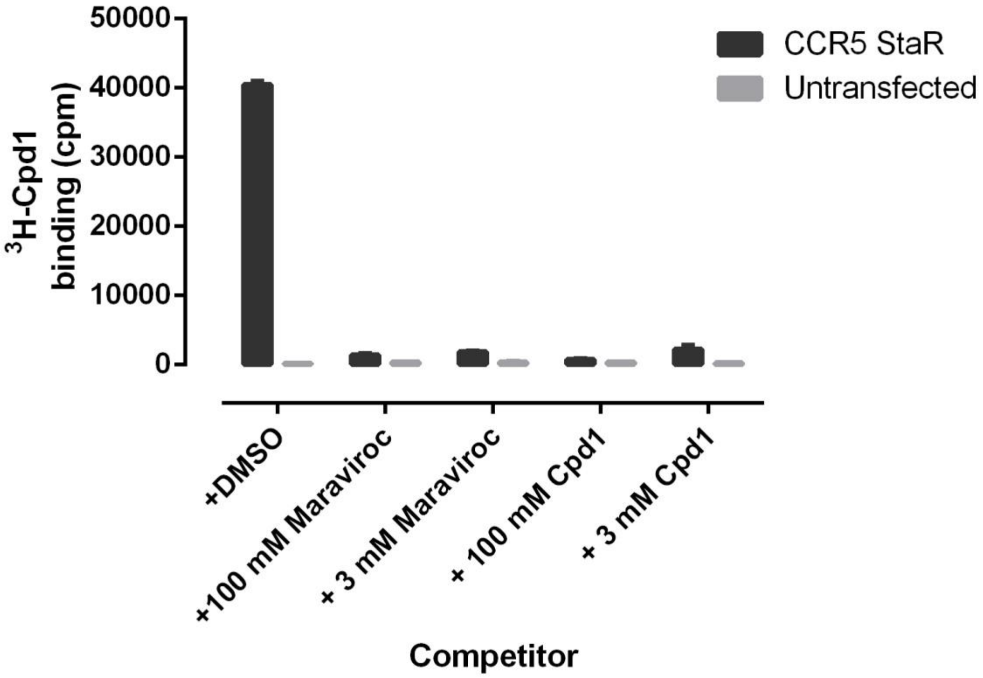

Figure S3. Theoretical shifts in the p- and s-polarized resonance angle and TIR angle caused by the addition of a layer at different distance from the last layer (silica) of the prism. The complex refractive index (n) and thicknesses (t) used were the following: Prism (BK7) : n=1.5151 ; Silver n=0.048+4.27i, t=50 nm ; Silica n=1.457, t=460 nm ; added layer n = 1.4 , t=5 nm ; aqueous buffer n=1.335. The thicknesses of the buffer and the prism are several orders of magnitude bigger than the thin layers, and were therefore deemed infinite.

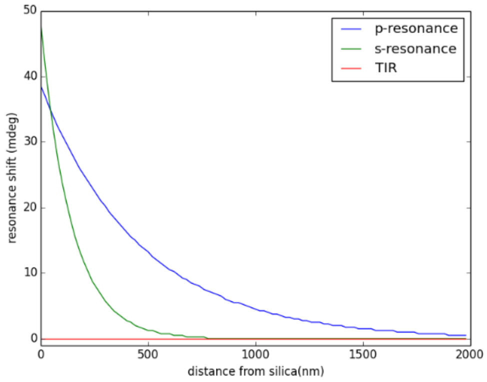

Figure S4. Ligand interaction with CCR5 StaR reconstituted in a POPC membrane monitored by PWR. CCR5 StaR was solubilised in DM and CHS and 1 μL of solution was injected in the PWR cell sample compartment containing the membrane. Maraviroc was incrementally added to the proteolipid membrane and the shifts in the resonance minimum position followed. The data was fitted with a hyperbolic binding equation that describes total binding to a single site in the receptor (more details in Materials and Methods). A dissociation constant of 5.2 nM was obtained.

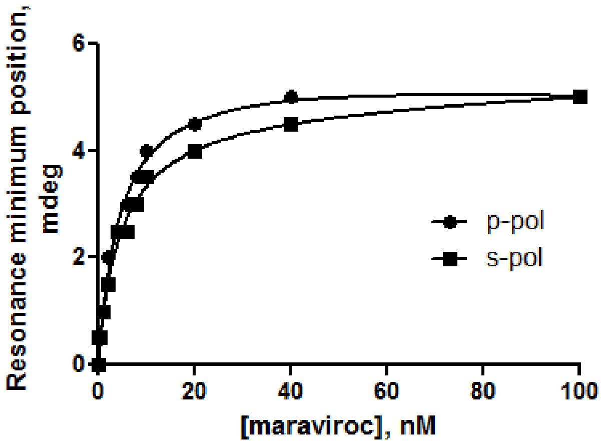

Supplement: Supplementary Information [file srep36181-s1.pdf]
